# Cholera Outbreaks in India, 2011–2020: A Systematic Review

**DOI:** 10.3390/ijerph19095738

**Published:** 2022-05-08

**Authors:** Basilua Andre Muzembo, Kei Kitahara, Anusuya Debnath, Ayumu Ohno, Keinosuke Okamoto, Shin-Ichi Miyoshi

**Affiliations:** 1 Graduate School of Medicine, Dentistry and Pharmaceutical Sciences, Okayama University, Okayama 700-8530, Japan; keikitahara@okayama-u.ac.jp (K.K.); anusuyadebnath@yahoo.co.in (A.D.); py386nyz@okayama-u.ac.jp (A.O.); okamot-k@okayama-u.ac.jp (K.O.); miyos-s@okayama-u.ac.jp (S.-I.M.); 2 Collaborative Research Center of Okayama University for Infectious Diseases in India, Kolkata 700010, India; 3 Department of Biotechnology, Brainware University, Kolkata 700125, India

**Keywords:** cholera, outbreak, water supply, open defecation, sewage, household, food, close contact, behavioral changes, India

## Abstract

Fecal contamination of water sources and open defecation have been linked to cholera outbreaks in India. However, a systematic review on the drivers responsible for these outbreaks has yet to be published. Here, we systematically review the published literature on cholera outbreaks in India between 2011 and 2020. We searched studies in English in three databases (MEDLINE, EMBASE, and Web of Science) and the Integrated Disease Surveillance Program that tracks cholera outbreaks throughout India. Two authors independently extracted data and assessed the quality of the included studies. Quantitative data on the modes of transmission reviewed in this study were assessed for any change over time between 2011–2015 and 2016–2020. Our search retrieved 10823 records initially, out of which 81 full-text studies were assessed for eligibility. Among these 81 studies, 20 were eligible for inclusion in this review. There were 565 reported outbreaks between 2011 and 2020 that led to 45,759 cases and 263 deaths. Outbreaks occurred throughout the year; however, they exploded with monsoons (June through September). In Tamil Nadu, a typical peak of cholera outbreaks was observed from December to January. Seventy-two percent (33,089/45,759) of outbreak-related cases were reported in five states, namely Maharashtra, West Bengal, Punjab, Karnataka, and Madhya Pradesh. Analysis of these outbreaks highlighted the main drivers of cholera including contaminated drinking water and food, inadequate sanitation and hygiene (including open defecation), and direct contact between households. The comparison between 2011–2015 and 2016–2020 showed a decreasing trend in the outbreaks that arose due to damaged water pipelines. Many Indians still struggle with open defecation, sanitation, and clean water access. These issues should be addressed critically. In addition, it is essential to interrupt cholera short-cycle transmission (mediated by households, stored drinking water and foodstuffs) during an outbreak. As cholera is associated with deprivation, socio-economic development is the only long-term solution.

## 1. Introduction

Cholera is a disease associated with destitution [1]. The heavy reliance on untreated environmental water sources for daily water needs such as drinking, bathing, cooking, and washing utensils by poverty-stricken communities increases the risk of ingesting copepods, the biotic carriers for cholera-causing bacteria *Vibrio cholerae* O1 or O139 (*V. cholerae*). Copepods soar in environmental water due to fluctuations in several climatic factors such as increased water temperature. Under such conditions, this increases the likelihood of ingesting an infective dose of *V. cholerae* through copepod-infested water [2]. Transmission of cholera spreads further upon contamination of drinking water sources or food with feces from infected people. Studies have demonstrated that host factors (such as age, nutrition, and blood group) also play a role in the development of cholera [3,4,5,6].

In October 2017, the WHO Global Task Force on Cholera Control (GTFCC) launched a vigorous fight against cholera. The GTFCC aims for the elimination of the disease as a public threat by 2030 in at least 20 countries with an emphasis on multiple targets including preventing the recurrence of cholera in hotspots [7].

Cholera outbreaks are relatively frequent in India. Surveillance data reveal a steady increase in reported cholera outbreaks throughout the country. From 1997 to 2006, 68 outbreaks were reported [8], while the reported outbreaks rose to 559 between 2009 and 2017 [9]. However, this is only the tip of the iceberg. The disease is grossly underreported in India [10], and despite these figures, cholera remains an under-recognized health issue in India [1,8]. Many state authorities are unaware of the disease burden and its impact on the citizens [11]. In India, cholera is endemic and occurs with marked seasonal dynamics; cholera is prevalent in the hot, humid and rainy season. In general, the seasonality of cholera outbreaks is mediated by various contributing and overlapping factors such as environmental parameters and climatic factors [2,12], waning host-immunity [13], and probably human behaviors (e.g., social gatherings) and activities also. For instance, the tribal communities (poorest and marginalized Indian communities) usually work in the paddy fields during the rainy season and became ill after drinking untreated environmental water [14]. Although access to safe drinking water and improved sanitation has been enhanced in most of the states and union territories (SUTs) by the *Swachh Bharat* (Clean India) Mission, substantial progress is still needed because of high inequities in distribution. For instance, only 16% of the population in rural India had access to piped water up until 2015 [15,16]. In addition, sanitation is another major hurdle to be handled. Approximately sixty percent of the world’s population who defecate in the open are in India. The overwhelming majority of individuals in rural India engage in open defecation that leads to the contamination of water bodies and heavy rainfall further worsens the situation [14,17]. As a remote driver of cholera, high rainfall raises the water level causing sewage and stagnant water to leak into damaged pipelines [14].

Several analyses of cholera outbreaks occurring in India have been documented [8,9,18,19]. However, the drivers of these outbreaks have yet to be systematically synthesized. The most recent report [9] summarized outbreaks from 2009 to 2017 and focused on antimicrobial resistance. Although this is only four years ago, data on fresh outbreaks have also become available. While it is useful and important in understanding antimicrobial resistance, the latter [9] did not address the importance of shifts in human behaviors in addition to access to clean drinking water to interrupt transmission during cholera outbreaks. Hence, both a timely update of data and a detailed synthesis of the evidence base for public health are warranted for policy recommendations.

The objective of this study is to provide the trend of cholera outbreaks in India over the last ten years. In addition, we also analyzed data for potential changes in the pattern of drivers of cholera. We specifically sought to understand whether there is any decreasing trend among the key drivers of cholera outbreaks by comparing two periods: 2011–2015 and 2016–2020. As cholera outbreaks are strongly seasonal in India, this makes us question what human-behavioral practices are associated with these seasonal fluctuations. We argue that broad shifts in behaviors are central to effective outbreak control. The data reviewed here would prove useful for informing policy-makers by pinpointing the areas where efforts should be focused for better prevention measures (such as motivating people in rural areas to use toilets, providing tap water to every household in the rural areas and urban slums, along with education on health and hygiene, education on household water storage, and mass availability of oral cholera vaccine for target-oriented vaccination), enhancing advocacy for launching a National Cholera Control Program in India or at least strengthening the sentinel surveillance system for diarrheal diseases and cholera in particular.

## 2. Methods

### 2.1. Data Sources

We undertook a systematic review according to the preferred reporting items for systematic reviews and meta-analysis (PRISMA) [20] to explore cholera outbreaks in India from the period 2011 to 2020. This systematic review is registered with PROSPERO (CRD42021233348). We defined a cholera outbreak as the occurrence of “at least one laboratory confirmed cholera case either by culture or polymerase chain reaction and there was evidence of local transmission in a specified geographical area or could be linked by place and time” [21]. For practical purposes, we considered a cholera outbreak as it had been defined in the studies included in this review.

We searched three electronic databases (MEDLINE through PubMed, EMBASE, and Web of science) for studies that were published on cholera outbreaks from January 2011 to December 2020 in India. This period of 10 years was chosen based on a similar review conducted by Kanungo and colleagues in which they also analyzed data over a ten-year period (1997–2006) [8]. The following groups of keywords were employed for cholera: “Cholera” OR “*Vibrio cholerae*” OR “*Vibrio cholerae* O1” OR “*Vibrio cholerae* O139”. These keywords were combined with: “outbreak” OR “epidemiology” OR “epidemics” OR “pandemic” OR “prevalence” OR “incidence” OR “risk factors”, OR “community” OR “immunity” AND “India.” We further narrowed down our searches by including each of the 36 names of SUTs. The search was further refined by scanning the reference lists of the obtained studies and related reviews. We did not apply any language restrictions during the search. Retrieved studies were exported to the Endnote software X9 (Clarivate, PA, USA) and duplicated studies were manually removed. Anticipating a scarcity of peer-reviewed publications on cholera outbreaks in India, the searches were supplemented by the grey literature data, i.e., the epidemiology reports of the Integrated Disease Surveillance Program (IDSP) that track cholera outbreaks [22]. The last search was performed on 6 April 2021. We used population, exposure, comparison, outcomes, and study design (PECOS) as a framework for study selection.

To be included, a study had to meet the following inclusion criteria:

(1) Population: any group of individuals affected by a cholera outbreak in India;
(2) Exposure: a study had to assess sources of exposure or potential risk factors for an outbreak;
(3) Comparison: it was not considered obligatory to include a comparison group for the present analysis;
(4) Outcomes: we focused on transmission routes as well as human practices that lead to cholera, the sources of the cholera outbreak and other human factors that may explain the seasonality of cholera;
(5) Study design: prevalence and incidence studies were eligible.

Articles were excluded for any of the following reasons: they were non-outbreak studies, reports were available in abstract form only, they investigated sporadic cholera cases, or the study failed to meet the above inclusion criteria.

### 2.2. Data Extraction and Analysis

Two authors independently screened articles for inclusion and abstracted data from the included studies. Disagreements were discussed and resolved by consensus. We devised a standardized chart to extract data. For each study, the extracted data included the first author’s last name, year of publication, setting and geographic region, duration of the outbreak, number of cholera cases, number of deaths, attack rates, investigated risk factors, behavioral characteristics of the index case, occurrence season, *V. cholerae* serogroup/serotype/biotype and required data for quality assessment. We also gathered data on two particular aspects related to the setting of each study: (1) urban versus rural, and (2) SUTs. Data on antibiotic resistance were also abstracted wherever applicable because antibiotic resistance is a serious public health issue that needs novel intervention strategies.

Data extracted from IDSP outbreak reports included information on setting, number of cases, number of deaths, date of onsets, and transmission vehicle.

Two authors independently assessed the quality of the included studies. The risk of bias in the included studies was assessed employing a modified Downes et al. appraisal checklist for cross-sectional studies [23].

Results were presented in both textual narrative and tabular formats. In addition, the geographical distribution of outbreaks was presented in area maps. The country area maps were generated using MapChart [24]. We generated graphs using the Stata software package (version 16, StataCorp LP, College Station, TX, USA). The prevalence of laboratory-confirmed cholera was synthetized using a random-effects model in the Comprehensive meta-analysis software, version 3. Annual reports of the Central Bureau of Health Intelligence (CBHI) on the national health profile of India were used to ascertain the Indian population by SUTs [25]. Cumulative cases were expressed as cholera cases per 100,000 persons. Quantitative data on the modes of transmission reviewed in this study were assessed for any change over time between the two time periods, i.e., period 1 (from 2011 to 2015) and period 2 (from 2016 to 2020).

## 3. Results

### 3.1. Study Characteristics

Overall, 10,823 records were identified initially, out of which 81 full-text studies were assessed for eligibility (Supplementary Figure S1). Among these 81 studies, only 20 met the inclusion criteria (Table A1). Most (90%; 18/20) of them were cross-sectional studies published from January 2011 to March 2021. All these 20 studies identified by our search strategy described 21 cholera outbreaks [26,27,28,29,30,31,32,33,34,35,36,37,38,39,40,41,42,43,44,45]. Cholera outbreaks were mostly reported in Southern and Eastern India (Table A1). In most studies, cholera diagnosis was often based solely on clinical symptoms, whereas laboratory confirmation of cholera was performed only in a limited number of patients (Table A2). The proportion of laboratory-confirmed cases ranged from 4.7% to 71.4%. The pooled detection rate of laboratory-confirmed cholera among suspected cases was 30.3% (95% confidence interval, 20.4–42.3; I^2^ = 88.4%) based on a random-effects meta-analysis of 15 studies (Table 1). The duration of these outbreaks ranged from 4 to 60 days (Table A1).

The majority (75%; 15/20) of included studies was scored as a moderate risk of bias, 25% (5/20) as a low risk of bias, and no study was deemed to have a high risk of bias (Table A3).

### 3.2. Geographical Distribution of Cholera Outbreaks

There were 565 outbreaks reported between 2011 and 2020 resulting in approximately 45,759 cholera cases and 263 (0.6%) deaths [22]. The median annual number of outbreaks reported during period 1 (2011 to 2015) was higher than period 2 (2016 to 2020). However, the difference was not statistically significant (66 versus 31; *p* = 0.058) (Table 2). In addition, the crude number of cases during 2011 to 2015 (*n* = 22,438; 49%) was lower compared with 2016 to 2020 (*n* = 23,321; 51%).

**Figure 1:**
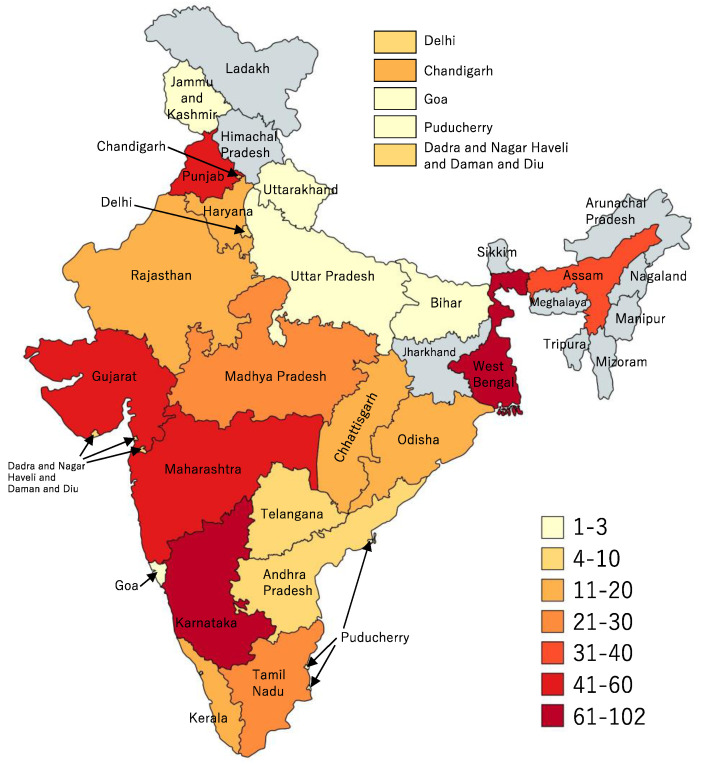
Cholera outbreaks (*n* = 565) by state and union territories, India, 2011–2020.

**Figure 2:**
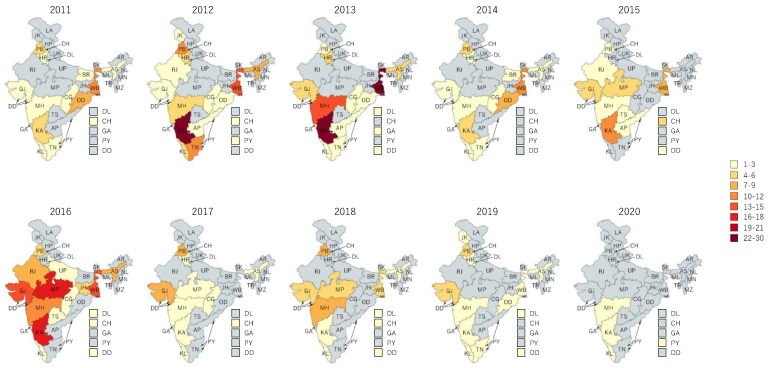
Cholera outbreaks (*n* = 565) by year and state, India, 2011–2020.

**Figure 3:**
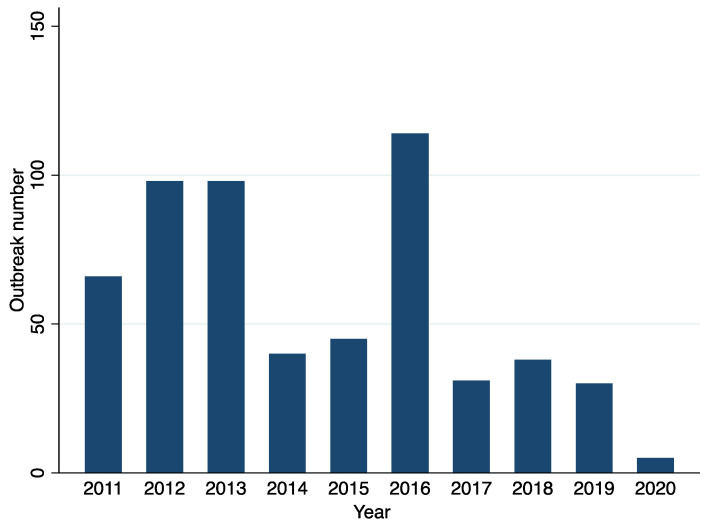
Cholera outbreaks (*n* = 565) by year, India, 2011–2020.

**Figure 4:**
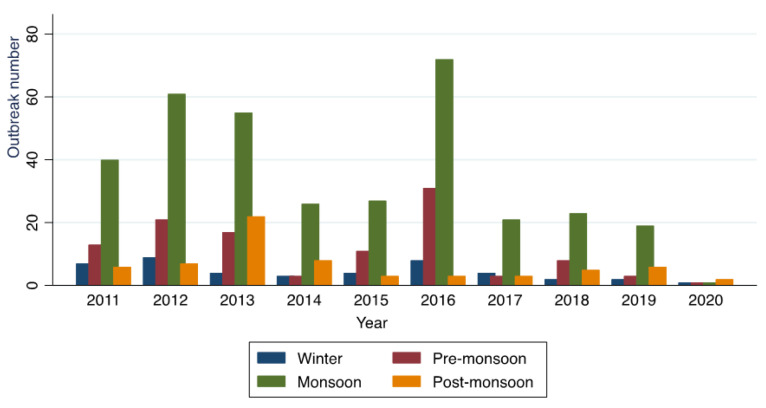
Cholera outbreaks (*n* = 565) by year and season, India, 2011–2020. Winter = December to January; Pre-monsoon = March to May; Monsoon = June to September; and Post-monsoon = October to November.

These outbreaks occurred in 24 of the 36 SUTs at least once between 2011 to 2020 (Figure 1). The occurrence of outbreaks varied greatly across the years. The highest number of reported outbreaks was recorded in 2016 (114/565; 20%), whereas the year 2020 had strikingly fewer (0.9%; 5/565) reported outbreaks than the previous years (Figure 2, Figure 3 and Figure 4). Five states, namely Gujarat, Karnataka, Maharashtra, Punjab, and West Bengal, reported a recurrence of cholera outbreaks every year from 2011 to 2019 (Figure 2). On the other hand, Karnataka and Maharashtra reported cholera outbreaks every year throughout the last 10 years that we have reviewed. When comparing period 1 (2011 to 2015) with period 2 (2016 to 2020), Delhi and Rajasthan reported cholera outbreaks during period 2 (2016 to 2020) but there was not a single report during period 1 (2011 to 2015) (Supplementary Figure S2).

Of the 565 outbreaks, Karnataka reported the most (102 outbreaks; 18%), followed by West Bengal (97 outbreaks; 17%), Maharashtra (54 outbreaks; 10%), Gujarat (53 outbreaks; 9%), Punjab (51 outbreaks; 9%), Assam (33 outbreaks; 6%), Madhya Pradesh (30 outbreaks; 5%), Tamil Nadu (25 outbreaks; 4%), and Odisha (19 outbreaks; 3%). The rest of the 27 SUTs reported 18% (101/565) of the outbreaks.

**Figure 5:**
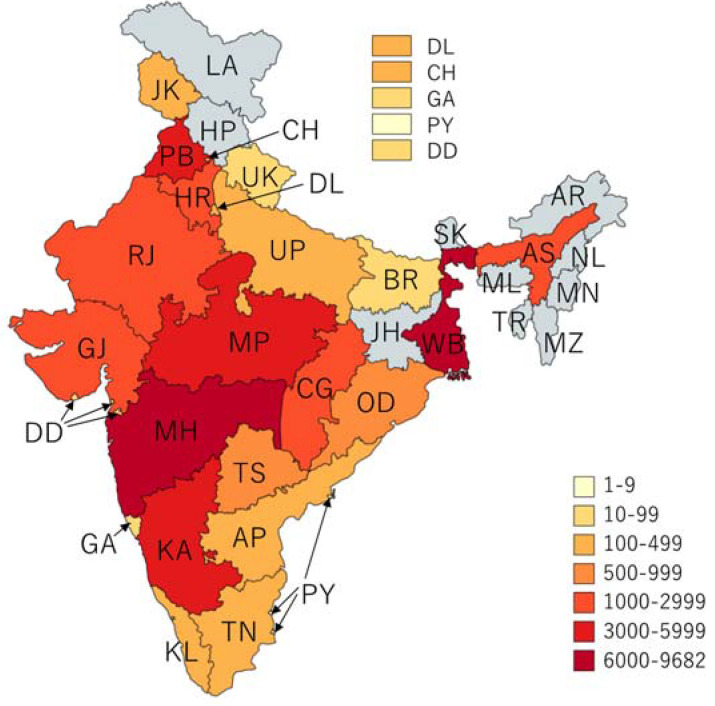
Reported cholera cases during outbreaks by state, India, 2011–2020.

**Figure 6:**
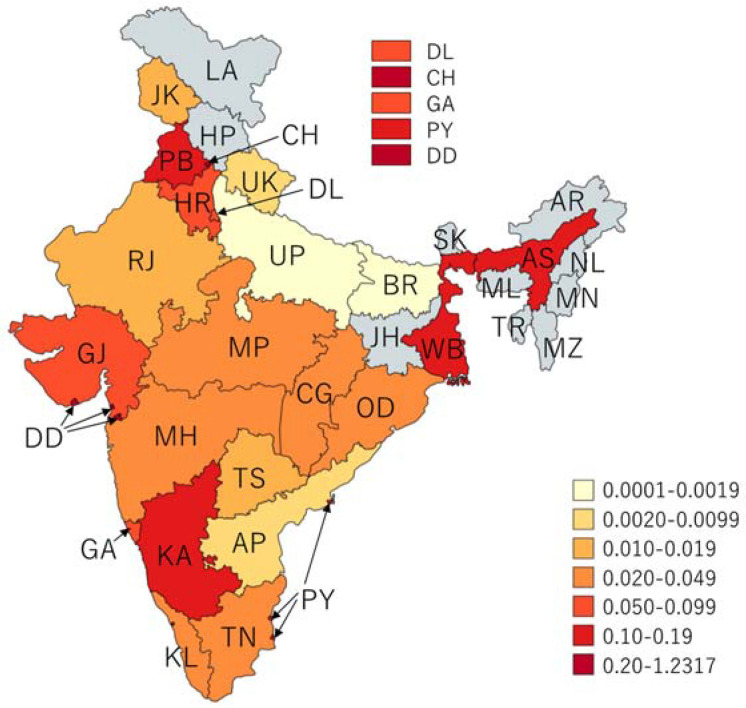
Rate of reported cholera outbreaks per 100,000 persons, India, 2011–2020.

**Figure 7:**
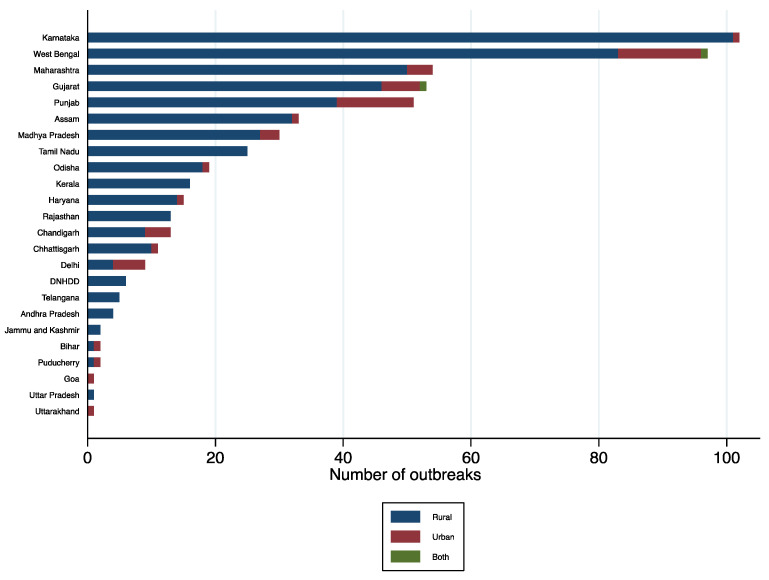
Cholera outbreaks (*n* = 565) by type of setting (rural vs. urban), India, 2011–2020. DNHDD = Dadra and Nagar Haveli and Daman and Diu.

The magnitude of outbreaks varied between the SUTs (Figure 5). Five states (Maharashtra, West Bengal, Punjab, Karnataka, and Madhya Pradesh) reported more than 3000 cases, which accounted for 72% of cases (33,089/45,759). The estimated incidence of cases during outbreaks remained low across the SUTs; the cumulative incidence was found to be the highest (1.2 cases per 100,000 persons) in the state of Chandigarh (Figure 6). Cholera outbreaks affected both rural and urban areas. However, 90% (507/565) of the outbreaks affected individuals living in rural areas (Table A1 and Figure 7), denoting that resuming progress towards cholera control in India needs increased efforts both in villages and urban slums.

### 3.3. Seasonality

**Figure 8:**
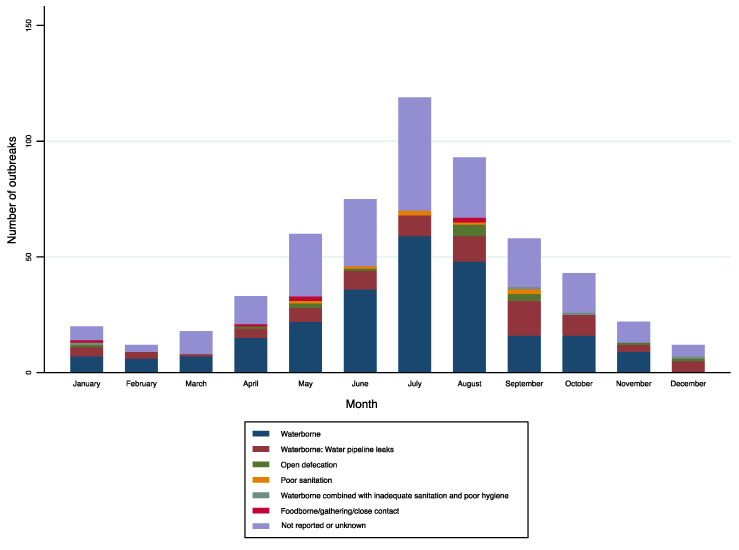
Number of cholera outbreaks (*n* = 565) by month and transmission routes, India, 2011–2020.

**Figure 9:**
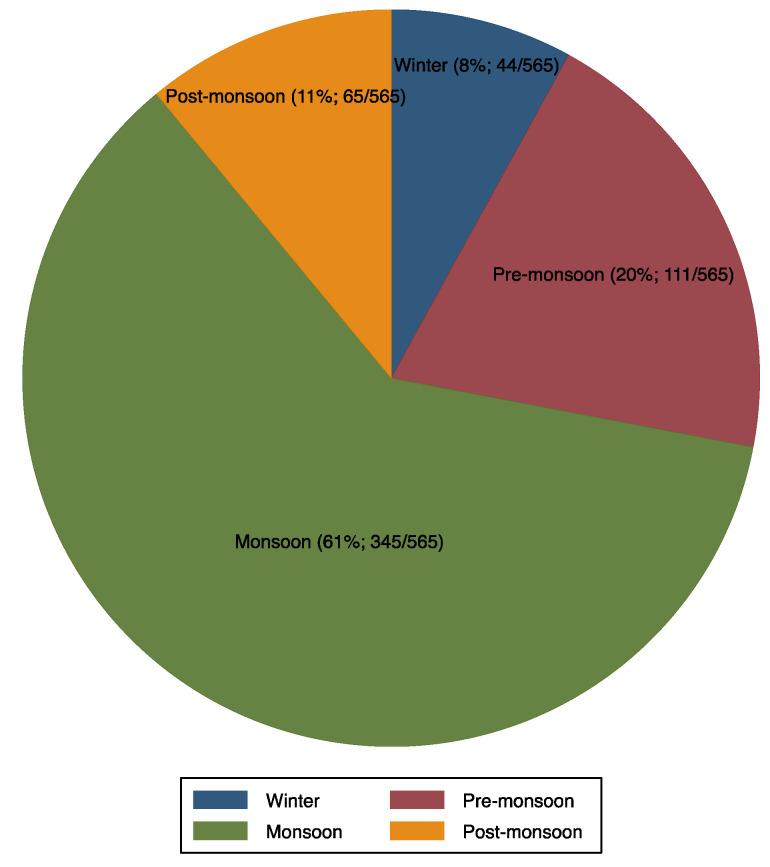
Cholera outbreaks (*n* = 565) in different seasons, India, 2011 to 2020. Winter = December to January; Pre-monsoon = March to May; Monsoon = June to September; and Post-monsoon = October to November.

Cholera outbreaks occurred throughout the year (Figure 4 and Figure 8); however, the explosion of outbreaks (61%, 345/565; Figure 9) occurred during monsoon season (June to September) in most of the SUTs and the peak was observed in July. The state of Tamil Nadu is the only exception, where the peak was observed during the winter season, from December to January.

### 3.4. Transmission Routes and Source of Water Contamination

From the IDSP surveillance data, the proportion of outbreaks in which the routes of transmission were identified was 62% (351/565), whereas 38% (214/565) had either unknown routes of transmission or were not reported. Among the 351 outbreaks, 319 (91%) transmission routes were the consumption of contaminated drinking water or exposure to unimproved water sources, and 32 (9%) were a lack of hygiene or inadequate sanitation.

**Figure 10:**
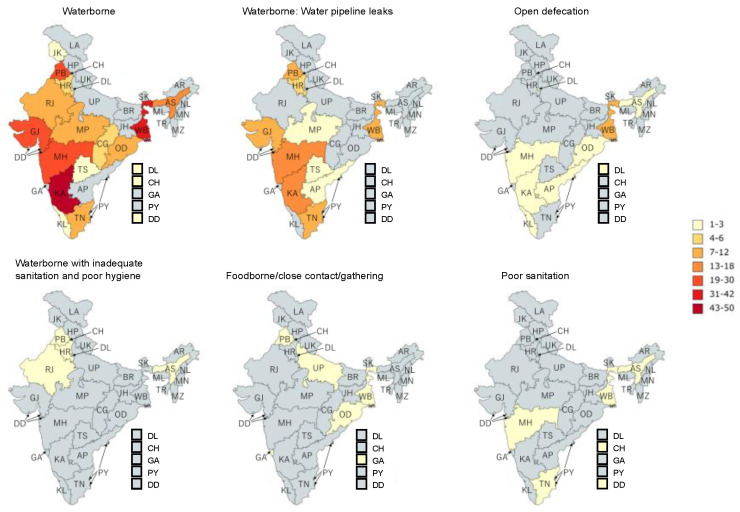
Number of cholera outbreaks (*n* = 565) by state and transmission routes, India, 2011–2020. Multiple modes of transmission were involved in some outbreaks.

**Figure 11:**
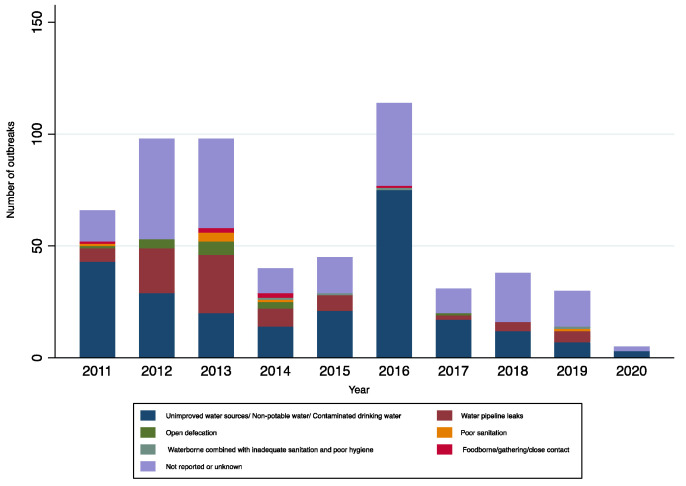
Number of cholera outbreaks (*n* = 565) by transmission routes and year, India, 2011–2020.

In more detail, transmission routes were (i) waterborne, including leaking water pipelines; (ii) inadequate sanitation or hygiene, including open defecation; (iii) waterborne with inadequate sanitation/hygiene; and (iv) foodborne/household spread or during social gatherings (Table 2; Figure 8, Figure 10 and Figure 11). As for changes over time in these transmission routes, a decreasing trend was observed in the number of outbreaks linked to leaking water pipelines (Figure 11). The median annual number of outbreaks due to leaking water pipelines from 2011 to 2015 was higher than from 2016 to 2020 (Table 2). However, there was no change in the median annual number of cholera outbreaks linked to other transmission routes, although the absolute number was generally higher from 2011 to 2015 (*n* = 347) compared with 2016 to 2020 (*n* = 218).

In some settings (Table A2), cholera outbreaks were specifically linked to the use of contaminated sources such as pond water [29,40], wells [26,28,41], pipe water [45], handpumps [32], leaky water pipelines [30,33,36,39,43,44,45], consumption of untreated municipal water [30], and unboiled water [39]. The one seasonal activity that could be linked to the cholera outbreaks was the period of paddy cultivation during which the farmers practice open defecation and consume drinking water from open wells within paddy fields [26] and from nearby rivers [35]. The spread of *V. cholerae* in India also benefits from the back-and-forth flow of the population for labor or trade between rural areas and peri-urban slums. For instance, when there is no work on the farms, seasonal waged labor drives rural people towards urban areas as part-time workers and thereafter these rural people return to the villages for farming during rainy seasons [35].

Several outbreaks were attributed to fecal contamination of drinking water, i.e., water samples with coliforms above the maximum permissible number [26,30,32,37,38,40,41,43].

Some outbreaks particularly arose in zones prone to natural disasters (i.e., flooding or cyclone) or during humanitarian emergencies as a result of water contamination due to overflowing toilets, canals, and drains [31], interrupted water distribution, or shortages of drinking water supply leading to the usage of unimproved water sources [34,38,39]. Shortage of drinking water during the summer also compelled people to use contaminated water [28].

However, it is sometimes challenging to isolate *V. cholerae* O1 or O139 from water samples. For instance, we noted that in a subset of studies, water samples were negative for *V. cholerae* even though patients showed typical cholera-like symptoms [32,33,35,41].

## 4. Discussion

In this study, we sought to understand whether there is any decreasing trend among the key drivers of cholera outbreaks in India by comparing two periods: 2011–2015 and 2016–2020. Of the reviewed modes of transmission (Table 2), only outbreaks due to damaged water pipelines showed a decreasing trend. As compared to a previous report summarizing cholera outbreaks from 1997 to 2006 [8], our review provides good evidence to substantiate the fact that access to safe water and sanitation continues to be an issue in India. A similar situation was also observed in Bangladesh, where leakages in water pipelines were the most frequent route of cholera transmission [46]. Damaged water pipelines and sanitation had also been hypothesized to spread cholera in Ghana, Guinea, and Sierra Leone [47]. Francois Jeannot recently pointed out that access to safe water and sanitation declined in Haiti from 1990 to 2015, and this issue creates a fertile ground for the spread of cholera [48].

From 2011 to 2020, we identified 565 reported cholera outbreaks that occurred every year. This is different from the African continent where outbreaks are sporadic in most African countries, except in some countries such as the Democratic Republic of the Congo (DRC) and Mozambique [49]. In 2016, cholera outbreaks were at their highest (Figure 3 and Figure 11). The reasons for this finding are unknown. While other explanations are possible, one hypothesis is that this increasing trend could have been the result of more thorough reporting of outbreaks from the affected SUTs. Another hypothesis is that India experienced its warmest year since 1901 in 2016 (ideal conditions for copepods to thrive). As a result, the amount of rain that fell during the 2016 monsoon varied, with below-normal rainfall in June and August (87%), and above-normal rainfall in July (107%; accompanied by flooding and cyclones), thus affecting water demands, especially for rural communities [50].

In this study, the overall number of cholera cases was lower (*n* = 45,759) compared with reported cases from 1997 to 2006 (*n* = 222,038) [8], but the case fatality rate was slightly higher during the period 2011–2020. The case fatality rate was 0.6% during the period 2011–2020 in contrast to 0.4% in the period from 1997 to 2006. However, the case fatality rate found in this study is within the range (0.07–0.6) reported in the previous ten-year period (1997 to 2006) [8]. Differences in the case fatality rate could be due to the current relatively improved surveillance and reporting in recent times.

The picture of cholera outbreaks has also changed in terms of geographical distribution. States with high morbidity were quite different in the recent decade (2011–2020) compared to the previous decade (1997 to 2006), except the state of West Bengal which consistently falls within the cholera-prone region. In this study, 72% of outbreak-related cases were reported from five states (Maharashtra, West Bengal, Punjab, Karnataka, and Madhya Pradesh). However, during the period 1997–2006, 91% of the cases were reported in four states (West Bengal, Odisha, Chhattisgarh, and Andaman and Nicobar Islands). This means that outbreaks are not limited only to the endemic states (such as West Bengal); thus, vigilance is needed even in states that do not report outbreaks.

Outbreaks were reported from 24 of the 36 SUTs. Despite having similar socio-economic difficulties in 12 no-cholera outbreak reporting states, this is a very unlikely scenario. This seems to be due to a general stigma against cholera in Indian society. This precludes the authorities from disclosing cholera outbreaks as it portrays a tarnished image of the water distribution networks and sanitation systems of their states [11]. Alternative explanations for underreporting could be attributed to the limited laboratory diagnostic resources, especially in the peripheral healthcare centers, along with constraints in cholera surveillance resources [1,8].

Only 21 cholera outbreaks were found in the peer-reviewed literature; an obvious explanation for this relatively low number of publications pertaining to the perceived notion of the investigators that this kind of outbreak reporting lacks novelty. Therefore, it is less likely to get published in a peer-reviewed journal [8]. Another explanation is that we might have missed some articles as Google scholar and Indian medical journals were not searched; we consider this to be one of the limitations of this study.

Despite the efforts of the Indian government to invest in efficient programmatic water sanitation and hygiene (e.g., *Swachh Bharat* Mission), there are numerous challenges to cope with, such as in-house contamination of drinking water [51], inadequate water infrastructures resulting in contamination of drinking water, and a shortage of water supply compelling people to use unimproved water sources. The fact that water was found to be the major vehicle for cholera outbreaks is not surprising because 90% (507/565) of reported outbreaks occurred in rural India, where inequity in clean water distribution is a significant problem. For example, in rural India, only 16% of people used improved piped water for drinking in 2015 [15,16]. In addition, the widespread fecal contamination of drinking water is still common in the country [26,30,32,37,38,40,41,43], in part due to higher rates of open defecation across the country and decaying sewage infrastructure.

Fecal contamination of the surrounding environment by persons infected with *V. cholerae* is frequently observed in India. This can be seen in the state of Odisha, where tribal people practice open defecation [29]. Another set of people who might be responsible for the fecal contamination is daily workers—people who move day-by-day to earn their living such as street vendors, farmers, fishermen, and traders. These people may be compelled either to practice open defecation or defecate in unimproved toilets in heavily polluted environments [52]. Therefore, a hygienic sanitation campaign for these people might serve a bigger purpose. It is also increasingly evident that exposure to *V. cholerae* in the country has centered overwhelmingly around some workplaces such as tea gardens, urban slums, and colonies where marginalized people of society reside due to a lack of access to basic water and sanitation services [19]. This observation is quite similar in countries where *V. cholerae* thrives. For instance, a fishing community in Uganda practices open defecation leading to cholera outbreaks [53] or discharges pit latrines into open drainage channels during heavy rains, contaminating well water, which also results in cholera outbreaks [54].

The studies reviewed suggest that *V. cholerae* can be transmitted through close person-to-person contact and also via environmental water during outbreaks In India. In contrast, *V. cholerae* is rarely detected in environmental water bodies of African countries (except some countries such as Mozambique). The principal mode of cholera spread was person-to-person contact in most African countries such as Uganda and Cameroon [49].

We found that contact with a patient suffering from cholera or an asymptomatic human carrier increased the risk of illness [37,40,55,56,57,58]. This may occur via fomites, food, or water (e.g., stored in-house water) contaminated with *V. cholerae.* Someone who touched infected fomites with *V. cholerae* unknowingly became a carrier, and in the absence of handwashing with soap, this carrier might, in turn, contaminate edibles or infect the person through a fecal–oral pathway. For instance, in-house fecal contamination of stored water represents a major hygiene problem in India. This issue was highlighted in one study where they found that 7% of stored water samples contained *V. cholerae* in the urban slums of Kolkata and 58% of samples had fecal coliforms higher than permissible limits [51]. This reminds us that we should not underestimate the basic health-promoting behavior of frequent handwashing with soap, especially in the context of India, due to two cultural habits. One of them is the habit of anal cleansing with water after defecation using hands and another one is eating with bare hands as socio-cultural norms. The lack of handwashing after anal cleansing followed by food consumption using those hands establishes an easy route for coliform intake. Households with limited access to handwashing resources (soap and running water) would not be able to often wash their hands and handwashing will less likely to be a priority and thus, the awareness about handwashing would be meaningless. Therefore, we need to develop and maintain hand-washing facilities alongside providing logistics to support hand-washing. Even the ample availability of handwashing facilities will neither automatically translate into their higher usage (high uptake) nor into effective health benefits because it requires substantial behavioral changes that might be difficult to maintain over time. Thus, we stress targeting educational efforts that would probably give desirable outcomes along with social mobilization, support for behavioral change and counselling as an alternative intervention strategy to enhance compliance in order to reduce exposure to *V. cholerae*.

During cholera outbreaks, cooking stations, areas in close proximity to the patient’s bed, and toilet floors were found to be the most contaminated surfaces in a household [59]. The sanitization of household surfaces and drinking water with chlorine-based disinfectant not only reduces cholera transmission but can also provide room for hygiene promotion. Therefore, it would be an ideal tool for curbing the burden of cholera. However, previous attempts to use household sprays to control cholera outbreaks did not warrant whether the procedure was effective because often this is not conducted in a timely manner, i.e., when *V. cholerae* had already been transmitted to other healthy household members by the sick person. It should be borne in mind that there are drawbacks associated with household spraying such as stigma, and household disinfection by a response team might increase hesitation among people to report cholera cases. Hence, new research is needed to yield sufficient evidence to support the use of household spraying during cholera outbreaks.

Countries such as Thailand [60] and Singapore [61] have also experienced contamination of food as the mode of cholera transmission, as with India. The consumption of contaminated food supplies remains a prominent driver of cholera outbreaks across SUTs, demonstrating that food-related transmission plays a non-negligible role in the spread of *V. cholerae* and we have to increasingly recognize the need to tackle this issue in order to ensure successful control of outbreaks. However, food-related cholera outbreaks have been under-explored in India with only very few published studies, which denotes a critical research gap. Any food contaminated with *V. cholerae* can spread the disease. In India, different foods had been incriminated in cholera outbreaks such as fermented rice, known as Pantha Bhat [40], milk products [56], and ice cream [62]. Some of these contaminated foods were from street vendors [40,62], indicating that food-related cholera outbreaks could still be a great public issue. Thus, the intervention methods targeting street food might be an effective method to prevent secondary transmission. Another critical factor is the presence of asymptomatic cholera carriers among the general population. These people, in spite of infection with *V. cholerae,* might remain asymptomatic but shed the bacteria in their feces and, therefore, are likely to sustain the transmission chain. This observation emphasizes the importance of targeting asymptomatic food handlers such as street food vendors by the investigators of outbreaks whenever food is suspected to be the cause of the outbreak. Especially, food handlers with diarrhea should be given advice on hygiene, and should not handle food that other people would eat. The observation of this review is consistent with the findings from a recent meta-analysis which reported that the consumption of street food was associated with a 5-fold increase in the odds of cholera [4]. These observations advocate for prevention efforts focused on tailored hygiene and cooking practices in people responsible for preparing food. In addition, advice must be given about the proper storage of cooked food and, if bound to keep food at ambient temperature because of poor resources, food must be heated before consumption. In some instances, it had been observed that uncontaminated food was unknowingly mixed with contaminated water due to a particular kind of food habit. This was illustrated in an outbreak triggered among villagers due to the consumption of fermented rice that was made using pond water that had neither been boiled nor chlorine treated. Even after villagers became ill, they said that the fermented rice tasted good only when pond water was used for its preparation [40], which further justifies the need to encourage behavioral changes as part of the prevention efforts.

There are two relevant limitations in the interpretation of our findings. Firstly, the depiction of our conclusion is based on the cholera outbreak data provided by the IDSP surveillance system and peer-reviewed articles which most likely underestimate the number of cholera outbreaks that have occurred in India since 2011. One possible explanation for this probable underestimation is that many outbreaks were classified as of unknown etiology and recorded in IDSP as outbreaks of acute watery diarrhea [22] and some outbreaks could have been missed during the literature search. Secondly, there were differences in outbreak notifications over time periods or SUTs. This means that for any comparisons of trends, one needs to apply caution in interpreting the data of interferences that could influence the detection of outbreaks along with their reporting systems. Notably, the decreased number of cholera outbreaks reported in 2020 was likely due to constraints in surveillance because of the COVID-19 pandemic, and SUTs with viable IDSP infrastructure and diagnostic facilities were more likely to report more about cholera outbreaks as compared to other SUTs with rudimentary surveillance structures [9].

## 5. Conclusions

In conclusion, an analysis of reported cholera outbreaks in India reconfirms that cholera is indeed a disease associated with destitution which mostly affects the neglected population. Most of the outbreaks occurred in rural India, where only 16% of people used improved piped water for drinking and open defecation is a common practice. Surprisingly, outbreaks due to damaged water pipelines showed a decreasing trend when a comparison was made between the two time periods 2011–2015 and 2016–2020.

Cholera outbreaks in India are likely to recur unless social and economic development (including higher education and better housing) improves dramatically along with the termination of apparently insurmountable behaviors such as doing the laundry in ponds, hygienic bathing in an environmental water source after defecation, open defecation, infrequent handwashing, and eating unhygienic street foods. The spread of *V. cholerae* during outbreaks should not be interrupted only through the intrusion of long-cycle transmission (mediated through the environment and water supply) but also by the interruption of short-cycle transmission of cholera mediated by unhygienic practices of households and food contamination.

Previous studies investigating outbreaks in India have recommended equally important measures that can be applied to counter future outbreaks. These include targeted use of cholera vaccines, access to safe drinking water, chlorination of water sources, regular disinfection of tube wells and wells, filtering the water with a piece of silk cloth, supplying oral rehydration salts (ORS), antibiotics and bleaching powder, use of telemedicine, action research, adequate sanitation, promotion of good personal hygiene, education and awareness campaigns (e.g., regarding latrine sanitation), safe food handling, proper sewage disposal, construction of drainage water away from the water pipelines, and long-term disease surveillance.

## Figures and Tables

**Table 1:** Pooled prevalence of laboratory-confirmed cholera during outbreaks (India, 2011–2020).

Study	Number of Stool Samples Examined	Number of Positive Samples	Detection Rate, % (95% CI)	Weight (%)
Dutta, 2021 [26]	34	11	32 (19 to 50)	7.7
Jain, 2021 [27]	18	4	22 (9 to 47)	6.4
Kale, 2020 [28]	711	109	15 (13 to 18)	8.9
Nayak, 2020 [29]	65	27	42 (30 to 54)	8.4
Singh, 2020 [30]	129	6	5 (2 to 10)	7.4
Mukhopadhyay, 2019 [31]	204	63	31 (25 to 38)	8.8
Goswami, 2019 [32]	28	2	7 (2 to 25)	5.4
Gopalkrishna, 2019 [33]	46	6	13 (6 to 26)	7.3
Pal, 2019 [34]	20	15	75 (53 to 89)	6.8
Pal, 2017 [35]	17	11	65 (40 to 83)	6.8
Allam, 2015 [38]	10	1	10 (1 to 47)	3.8
Bhattacharya, 2015 [37]	6	4	67 (3 to 92)	4.7
Fredrick, 2015 [39]	16	9	56 (32 to 78)	6.9
Dey, 2014 [41]	7	5	71 (33 to 93)	4.8
Mahanta, 2013 [45]	13	3	23 (8 to 52)	5.9
Total (random effects)	1324	276	32 (23 to 44)	100.0

Definition of abbreviation: CI = confidence interval.

**Table 2:** Number of cholera outbreaks during the period 2011–2015 compared with 2016–2020.

Transmission Routes	Number of Outbreaks during 2011–2015, *n* (%)	Number of Outbreaks during 2016–2020, *n* (%)	Median (Min-Max) Annual Outbreaks Number during 2011–2015 versus 2016–2020	*p* Value *
Unimproved water sources/Non-potable water/Contaminated drinking water	127 (36.6)	114 (52.3)	21 (14–43) vs. 12 (3–75)	0.058
Water pipeline leaks	67 (19.3)	11 (5.0)	8 (6–26) vs. 4 (2–5)	**0.028** **
Open defecation	14 (4.0)	1 (0.5)	4 (1–6) vs. 1 (1–1)	0.361
Poor sanitation	6 (1.7)	1 (0.5)	1 (1–4) vs. 1 (1–1)	0.505
Waterborne combined with inadequate sanitation and poor hygiene	2 (0.6)	2 (0.9)	1 (1–1) vs. 1 (1–1)	-
Foodborne/gathering/close contact	5 (1.4)	1 (0.5)	2 (1–2) vs. 1 (1–1)	0.248
Not reported or unknown	126 (36.3)	88 (40.4)	16 (11–45) vs. 16 (2–37)	1.000
Total	347 (100.0)	218 (100)	66 (40–98) vs. 31 (5–114)	0.058

* *p* values were calculated using Fisher’s exact test. They are comparing the median annual outbreaks number during 2011–2015 versus 2016–2020. ** *p* value < 0.05.

## Data Availability

All relevant data are within the manuscript and its supporting information files.

## Supplementary Materials

The following supporting information can be downloaded at: https://www.mdpi.com/article/10.3390/ijerph19095738/s1, Supplementary Figure S1: Flow chart showing evidence search and selection of studies (Cholera outbreaks in India, 2011–2020); Supplementary Figure S2: Cholera outbreaks in selected state and union territories comparing 2011–2015 with 2016–2020. The states of Tamil Nadu and Haryana did not cholera outbreaks during 2016–2020.

## Appendix A

**Table A1:** Characteristics of included studies.

Outbreak Number	References	Location	Urban/Rural Area	Study Design/Type	Study Period	Age (Year)/Descriptor	Outbreak Duration (Days)	Population at Risk	Number of Cholera Cases	Attack Rate (Case/100 Person)	Case Fatality Ratio (Number of Death)	Occurrence Month
1	Dutta, 2021 [26]	Ghughri, Madhya Pradesh, Central	Rural	Cross-sectional	2016	27 (1–76)	30	101,115	628	0.6	2 (14/628)	August
2	Jain, 2021 [27]	Shahpur, Haryana, Northern	Rural	Cross-sectional	2019	18 (1–65)	29	2602	196	8	1 (2/196)	September
3	Kale, 2020 [28]	Yavatmal, Maharashtra, Western	Rural	Cross-sectional	2018	All	-	-	-	-	-	March–July
4 and 5	Nayak, 2020 [29]	Odisha, Eastern	Rural	Cross-sectional	2018	>5	4	1387	55	4.0	0	August
					2019	>5	5	500	73	14.6	1.4	April
6	Singh, 2020 [30]	Bhadola, Delhi, Northern	Urban	Case-control	2018	Median = 14.5	56–59	7280	129	1.8	0	April-May
7	Mukhopadhyay, 2019 [31]	Kolkata and vicinity, West Bengal, Eastern	Urban	Cross-sectional	2015	Median = 26	15	-	-	-	1 death	August
8	Goswami, 2019 [32]	Wardha, Maharashta, Western	Urban	Cross-sectional	2018	3–65	9	104	28	27	0	July
9	Gopalkrishna, 2019 [33]	Aurangabad, Maharashta, Western	Urban	Cross-sectional	2017	>14 (90%)	12	16,000	7447	47	-	November
10	Pal, 2019 [34]	Odisha, Eastern	Rural	Cross-sectional	2018	All	-	-	-	-	0	May
11	Pal, 2017 [35]	Narla, Kalahandi, Odisha, Eastern	Urban	Cross-sectional	2014	>20	60	46,236	321	0.7	0.9	July–September
12	Uthappa, 2015 [36]	Medipally, Telangana, Southern	Rural	Case-control	2013	All	9	–	138	11.5	0.7(1 death)	November
13	Bhattacharya, 2015 [37]	Somanakoppa, Bagalkot, Karnataka, Southern	Rural	Cross-sectional	2013	-	12	–	49	3.5	–	August
14	Allam, 2015 [38]	Medak, Andhra Pradesh, Southern	Rural	Cross-sectional	2013	All (0–74)	30	281		3.3	1.4 (3 deaths)	August
15	Fredrick, 2015 [39]	Pondicherry, Puducherry, Southern	Urban	Case-control	2012	All	13	8367	921	11	0.1 (1 death)	January
16	Biswas, 2014 [40]	Haibatpur, West Bengal, Eastern	Rural	Cross-sectional	2012	33 (5 to 80)	14	780	41	5	0	June
17 and 18	Dey, 2014 [41]	Talikoti, Bijapur, Karnata, Southern	Semi-rural	Cross-sectional	2012	All	20	26,205	101	0.4	0	July–August
		Harnal, Bijapur, Karnata, Southern	Rural	Cross-sectional	2012	All	7	960	200	21	0	July–August
19	Kumar, 2014 * [42]	Kalamb and Yavatmal, Maharashtra, Western	Urban	Cross-sectional	2012	-	-	-	-	-	4.5	May
19	Kumar, 2014 * [43]	Kalamb and Yavatmal, Maharashtra, Western	Urban	Cross-sectional	2012	-	-	-	-	-	-	May
20	Puri, 2014 [44]	Vikas Nagar, Chandigarh, Northern	Urban	Cross-sectional	2012	All	14	15,000	1875	15	(4 deaths)	July
21	Mahanta, 2013 [45]	Bagjan, Sivasagar, Assam, North-eastern	Rural	Cross-sectional	2012	41 (3–70)	-	2503	120	4.8	0.83 (1 death)	May

*: These two studies described the same outbreak.

**Table A2:** Sources of outbreaks.

Study	Risk Factors Assessed	Men (%)	Women (%)	Population	Cholera Definition	Serogroup	Serotype/Biotype	Transmission Route/Suspected Exposure	Number Examined	Number of Infected Individuals	Prevalence (95% CI)	Comment
Dutta, 2021 [26]	Water	39	61	Community	Clinical; Culture-confirmed	VC O1	Ogawa biotype El Tor	Contaminated drinking water	34	11	32	More women were affected.
Jain, 2021 [27]	Water, Environment	46	54	Community	Clinical; Culture-confirmed	-	-	Contaminated drinking water	18	4	22	Attack rates were highest in the 11–20 years group
Kale, 2020 [28]	None	-	-	-	Clinical; Culture-confirmed	VC O1	Ogawa biotype El Tor	Contaminated drinking water	711	109	15	Males and women were equally affected
Nayak, 2021 [29]	Water, Hygiene	-	-	-	Clinical; Culture-confirmed	Haitian variant of VC O1	Ogawa biotype El Tor	Pond water used to cook foods and clean utensils at a local festival and marriage ceremony	65	27	42 (30 to 54)	Children < 5 were not affected. More women were affected
Singh, 2020 [30]	Water, Hygiene, Knowledge on diarrhea transmission	48	52	-	Clinical; Culture-confirmed	VC O1	Ogawa biotype El Tor	Drinking untreated municipal water	129	6	5 (2 to 10)	-
Mukhopadhyay, 2019 [31]	Habitation	56	44	Hospital-based surveillance	Clinical; Culture-confirmed	VC O1	Ogawa biotype El Tor and Inaba	Living near water channel and central lake channel. Contamination of drinking water sources due to overflowing of canals and drains during heavy rains	204	63	31 (25 to 38)	Age range: 5 months to 99 years. No difference between men and women
Goswami, 2019 [32]	Habitation location, Water	-	-	-	Clinical; Culture-confirmed	VC O1	Ogawa biotype El Tor	Hand pump; drinking water	28	2	7 (2 to 23)	Most cases were children (0–10); More males were affected
Gopalkrishna, 2019 [33]	Water	-	-	-	Clinical; Culture-confirmed	VC O1	Ogawa biotype El Tor	Fecal contamination of the river water and leakage in the pipeline	46	6	13 (6 to 26)	-
Pal, 2019 [34]	Water	-	-	-	Clinical; Culture-confirmed	VC O139	-	Heavy rain contaminated muddy water supply	20	15	75 (53 to 89)	-
Pal, 2017 [35]	Water	-	-	-	-	-	Ogawa biotype, ctxB7 variant of Haitian VC	Contaminated drinking water source, unhygienic conditions in the house, unsafe disposal of fecal materials, cleaning of excrement-contaminated clothes in nearby water reservoirs, visiting choleric patients	17	11	65 (41 to 83)	Prevalence high in children < 1 year
Allam, 2015 [38]	Water, Hygiene	-	-	-	Clinical; Culture-confirmed	VC O1	Ogawa biotype El Tor	Contaminated drinking water	10	1	-	-
Bhattacharya, 2015 [37]	Water, hygiene	-	-	-	Clinical; Culture-confirmed	VC O1	Ogawa biotype El Tor	Contaminated drinking water	6	4	-	-
Uthappa, 2015 [36]	Water, household size, hygiene, socio-demographics	53	47	-	Clinical; Culture-confirmed	VC O1	Ogawa biotype El Tor	Contaminated drinking water source	-	138	-	Prevalence high in children ≤ 5 year
Fredrick, 2015 [39]	Water, Hygiene	47	53	-	Clinical; Culture-confirmed	VC O1	Ogawa biotype El Tor	Contaminated drinking water	16	9	-	-
Biswas, 2014 [40]	Water, hygiene	69	31	-	Clinical; Culture-confirmed	VC O1	Ogawa biotype El Tor	Contaminated drinking water source	-	41	-	-
Dey, 2014 [41]	Water, Hygiene	-	-	-	Clinical; Culture-confirmed	VC O1	Ogawa biotype El Tor	Contaminated drinking water	7	5	-	All age-groups were affected
Kumar, 2014 [43]	Water, Environment	-	-	Hospital	-	VC O1	Ogawa biotype El Tor	Contaminated drinking water source	-	20	-	Leakage in water pipes mixing water with drainage
Puri, 2014 [44]	Water, Environment, Food, Mass gathering	53	47	Hospital and community	Clinical; Culture-confirmed	VC O1	Ogawa biotype El Tor	Contaminated drinking water source	-	8	-	-
Mahanta, 2013 [45]	Demographics, Socioeconomic, Environmental	-	-	-	Clinical; Culture-confirmed	VC O1	Ogawa biotype El Tor	Contaminated drinking water source	13	3	23	-

VC = vibrio cholerae; NR = not reported.

**Table A3:** Study quality.

Study	Aim Clearly Stated	Setting Provided	Study Design or Sampling Method Explained	Case Definition of Diarrhea or Cholera Clearly Mentioned	Statistical or Analysis Methods Reported	Risk Factors for Outbreak (or Causes of Outbreaks) Investigated	Case Fatality Ratio Reported	Performance of Confirmatory Test (Culture or PCR)	Limitations or Potential Confounders Discussed	Score	Risk of Bias
Dutta, 2021 [26]	Yes	Yes	No	Yes	Unclear	Yes	Yes	Yes	Yes	7	Moderate
Jain, 2021 [27]	Yes	Yes	No	Yes	Unclear	Yes	Yes	Yes	Yes	7	Moderate
Kale, 2020 [28]	Yes	Yes	No	No	No	Yes	Yes	Yes	No	5	Moderate
Nayak, 2020 [29]	Yes	Yes	Yes	Yes	No	Yes	Yes	Yes	No	7	Moderate
Singh, 2020 [30]	Yes	Yes	Yes	Yes	No	Yes	Yes	Yes	Yes	9	Low
Mukhopadhyay, 2019 [31]	Yes	Yes	Yes	Yes	No	Yes	Yes	Unclear	No	6	Moderate
Goswami, 2019 [32]	Yes	Yes	Yes	Yes	No	Yes	Yes	Yes	No	7	Moderate
Gopalkrishna, 2019 [33]	Yes	Yes	No	No	No	Yes	Yes	Yes	No	5	Moderate
Pal, 2019 [34]	Yes	Yes	No	No	No	Yes	Yes	Yes	No	5	Moderate
Pal, 2017 [35]	Yes	Yes	Yes	Yes	No	Yes	Yes	Unclear	No	6	Moderate
Uthappa, 2015 [36]	Yes	Yes	Yes	Yes	Unclear	Yes	Yes	Yes	Yes	9	Low
Bhattacharya, 2015 [37]	Yes	Yes	Yes	No	No	Yes	No	Yes	Unclear	5	Moderate
Allam, 2015 [38]	Yes	Yes	No	Yes	No	Yes	Yes	Yes	Yes	6	Moderate
Fredrick, 2015 [39]	Yes	Yes	Yes	Yes	No	Yes	Yes	Yes	Yes	9	Low
Biswas, 2014 [40]	Yes	Yes	Yes	Yes	Unclear	Yes	Yes	Yes	No	8	Low
Dey, 2014 [41]	Yes	Yes	Yes	Yes	No	Yes	Yes	Yes	No	7	Moderate
Kumar, 2014 [42]	Yes	Yes	Yes	Unclear	No	Yes	No	Yes	Unclear	5	Moderate
Kumar, 2014 [43]	Yes	Yes	Yes	No	No	No	Yes	Yes	Unclear	5	Moderate
Puri, 2014 [44]	Yes	Yes	Yes	Yes	No	Yes	Yes	Yes	Yes	8	Low
Mahanta, 2013 [45]	Yes	Yes	Yes	Yes	No	Yes	Yes	Yes	No	7	Moderate

A score “1” was given for each reported item. Scores were rated as having a low risk of bias (score of 8–9), moderate risk of bias (score of 5–7) or high risk of bias (score 4 or below). PCR: polymerase chain reaction.
