# Supplementary material for: Cholera Outbreaks in India, 2011–2020: A Systematic Review
Source: Int J Environ Res Public Health. 2022 May 8;19(9):5738. doi: 10.3390/ijerph19095738 (PMC9099871; doi:10.3390/ijerph19095738)

Supplementary Figure S1. Flow chart showing evidence search and selection of studies (Cholera outbreaks in India, 2011– 2020).

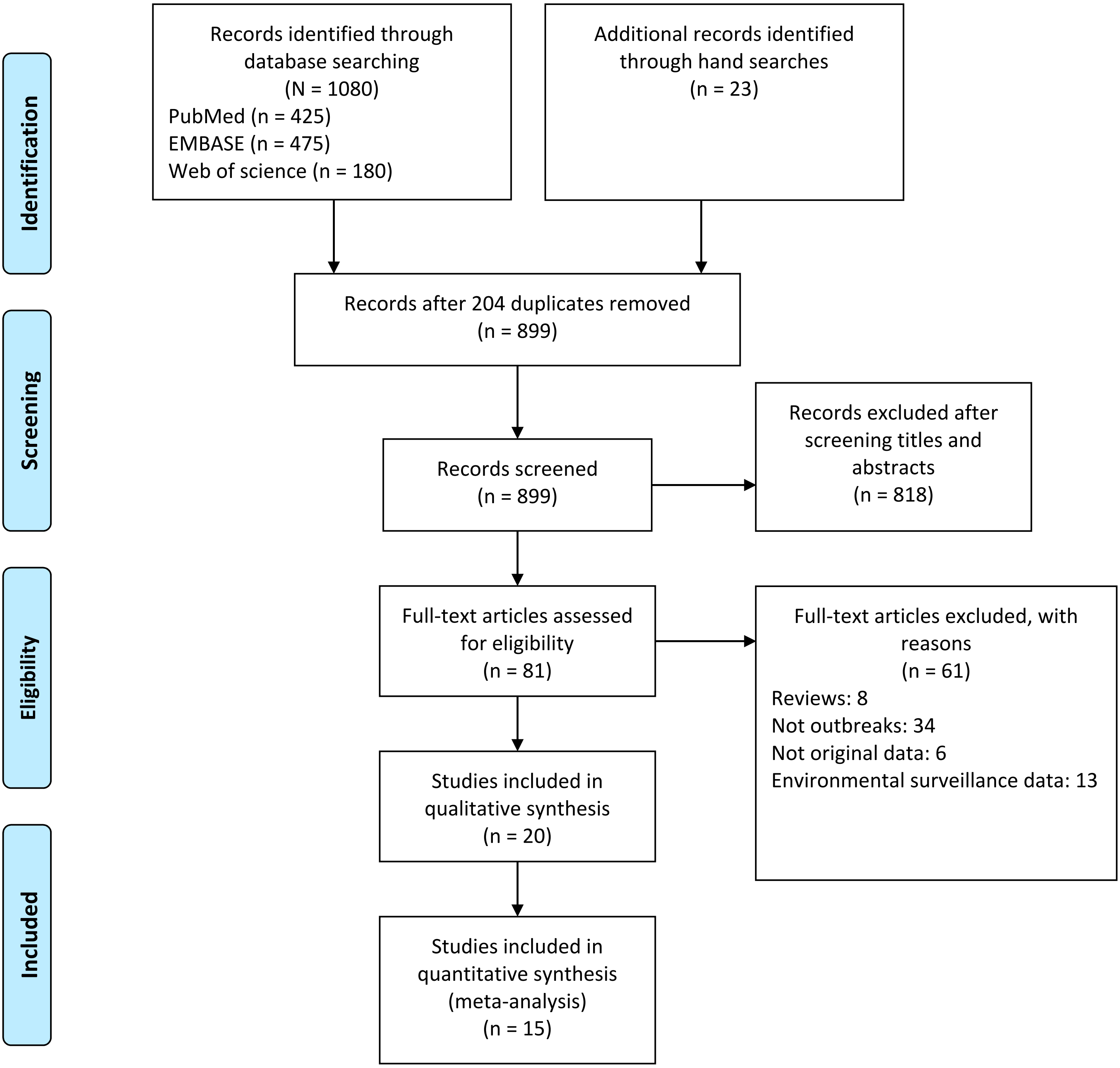

Supplementary Figure S2.
Cholera outbreaks in selected state and union territories comparing 2011-2015 with 2016-2020. The states of Tamil Nadu and Haryana did not cholera outbreaks during 2016-2020.

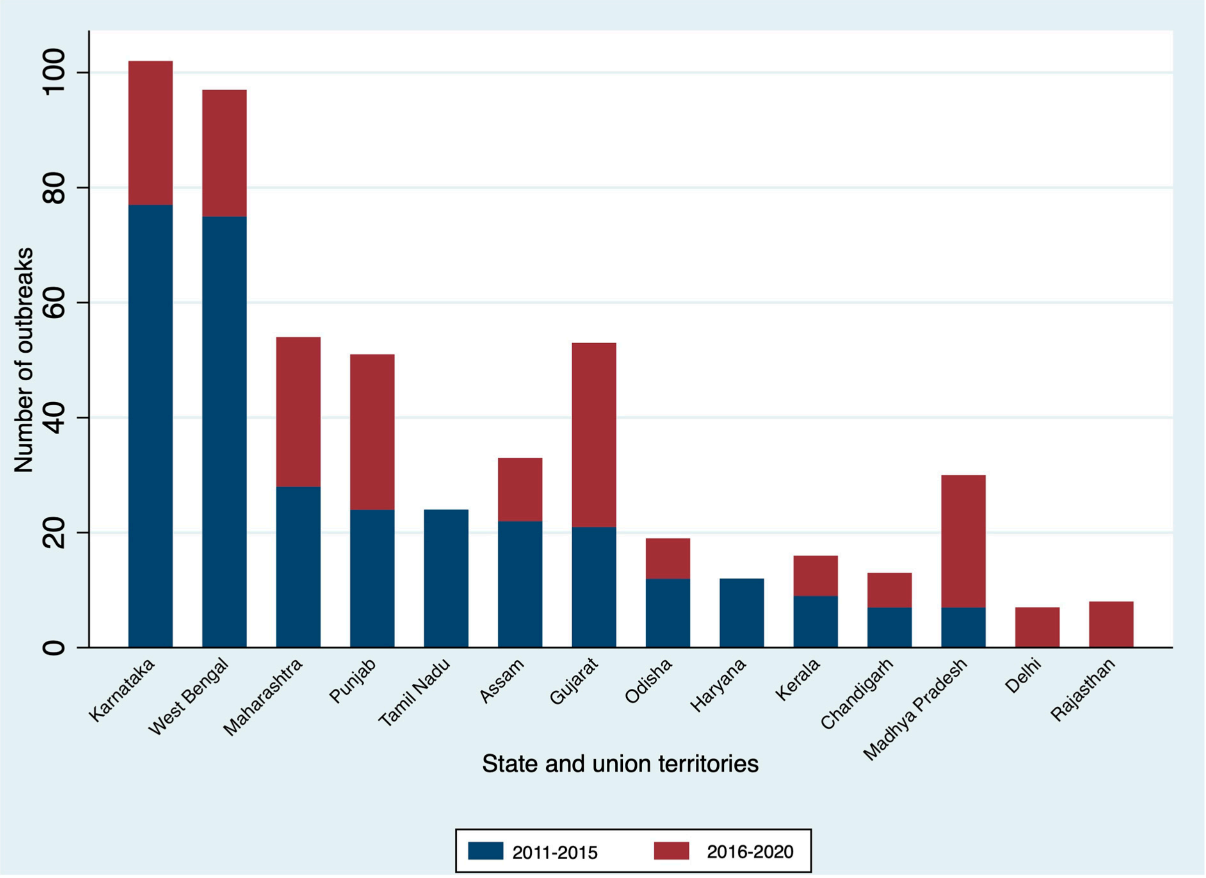

Supplement: Supplementary file 1 [file ijerph-19-05738-s001.zip › ijerph-1686518-supplementary.pdf]
